# Labyrinthitis Ossificans in a Post-Splenectomy Patient With Meningitis: A Case Report and Review of Literature

**DOI:** 10.7759/cureus.34555

**Published:** 2023-02-02

**Authors:** Nick Kamkari, Divya Chari

**Affiliations:** 1 Otolaryngology-Head and Neck Surgery, University of Massachusetts Chan Medical School, Worcester, USA; 2 Otolaryngology-Head and Neck Surgery, Massachusetts Eye and Ear, Boston, USA

**Keywords:** hearing rehabilitation, post-splenectomy, sensorineural deafness, cochlear implant, labyrinthitis ossificans

## Abstract

This report describes a case of cochlear implantation to treat profound deafness three months after a diagnosis of bacterial meningitis in a patient with a remote history of splenectomy. A 71-year-old woman with a remote history of a splenectomy over 20 years before presented with bilateral profound deafness that occurred as sequela from pneumococcal meningitis three months prior. The patient had been vaccinated against the 23-valent polysaccharide pneumococcal vaccine (PPV-23). The audiometric evaluation revealed no response in either ear. Imaging was suggestive of complete ossification of the right cochlea with partial ossification of the basal turn of the left cochlea. She underwent successful left-sided cochlear implantation. Standard post-implantation speech outcomes include consonant-nucleus-consonant (CNC) word and phoneme scores and Az-Bio in quiet and noise. The patient noted subjective improvement in her hearing. Performance measures markedly improved when compared to her pre-operative evaluation, which showed no aided sound detection. This case report highlights the possibility of meningitis many years after splenectomy that can result in profound deafness with labyrinthitis ossificans and the potential for hearing rehabilitation for cochlear implantation.

## Introduction

Asplenic individuals are known to be at an elevated risk for infection with encapsulated bacteria [1]. The incidence of overwhelming post-splenectomy infection (OPSI) is estimated to be approximately 0.18%-0.42% per year, with a lifetime risk of 5% [2,3]. Vaccination of post-splenectomy patients with pneumococcal polysaccharide, meningococcal, and Haemophilus influenzae type b vaccines is critical to avoid the neurologically devastating and potentially fatal complications of bacterial meningitis [4]. The increased risk of infection is related to the role of the spleen as a lymphoid organ that produces IgM memory B lymphocytes, which are crucial for the opsonization of encapsulated bacteria. In patients who are appropriately vaccinated, however, it is uncommon to develop meningitis or other associated complications [5,6].

A well-known neurological sequela of meningitis is hearing loss, affecting between 5%-33% of patients [7,8]. The severity of hearing loss can vary from mild to profound but is often bilateral, severe, and permanent, involving all frequencies. In response to the inflammatory and destructive process of meningitis, labyrinthitis ossificans can occur, resulting in pathologic new bone formation within the fluid-filled inner ear space of the otic capsule. Histopathologically, labyrinthitis ossificans are divided into three stages: acute, fibrous, and ossification [9]. Ossification can develop as soon as several weeks after acute bacterial meningitis. Recognition of this condition before consideration of cochlear implantation is critical, as an ossified cochlea presents challenges to surgical implantation of the cochlear implant (CI) electrode array.

Proactive vaccination is strongly recommended in post-splenectomy patients to mitigate the risk of morbidity and mortality associated with meningitis. While there does exist a risk of acquiring a serotype not prevented by the vaccine, in general, patients who follow vaccination guidelines and have antibody titers checked regularly are at reduced risk for severe infections [9,10]. Here, we present the case of a woman with a remote history of splenectomy with up-to-date vaccinations who developed pneumococcal meningitis, resulting in labyrinthitis ossificans and bilateral profound sensorineural hearing loss (SNHL) for which she underwent unilateral cochlear implantation.

A case report of a patient with a remote history of splenectomy who developed pneumococcal meningitis despite appropriate vaccination that resulted in profound bilateral sensorineural hearing loss with evidence of partial labyrinthitis ossificans is described, along with the relevant treatment and outcomes. A literature review of meningitis following splenectomy and surgical management of labyrinthitis ossificans with cochlear implantation was performed, and pertinent observations are summarized.

## Case presentation

**Figure 1:**
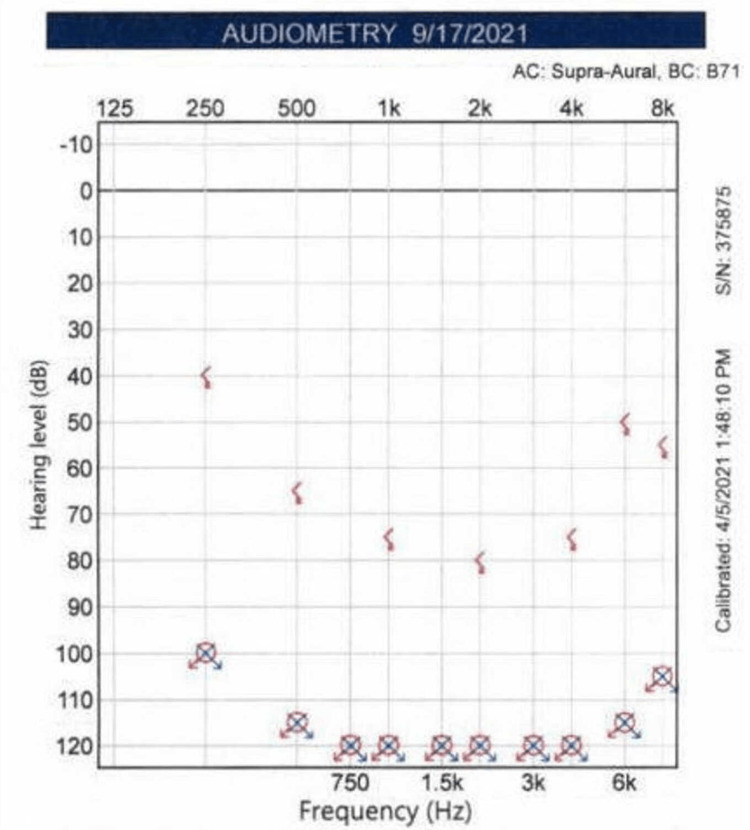
Pre-operative audiogram demonstrating bilateral profound deafness.

The patient presented at age 71, three months after developing profound bilateral sensorineural hearing loss from septic meningitis. She had a history of a splenectomy over 20 years prior for immune thrombocytopenia and had been treated with pneumococcal, meningococcal, and Haemophilus influenza (Hib) vaccines with careful monitoring of her antibody titers. Despite receiving the appropriate vaccinations, she presented to a local emergency department with fevers and malaise with blood cultures consistent with pneumococcus. The patient developed almost immediate hearing loss during her hospital stay but did not receive immediate audiology or otologic care. After a prolonged recovery from septic meningitis of three months, she was evaluated in an otology clinic. Upon presentation, her audiogram showed bilateral profound deafness with no discernable speech discrimination (Figure 1).

**Figure 2:**
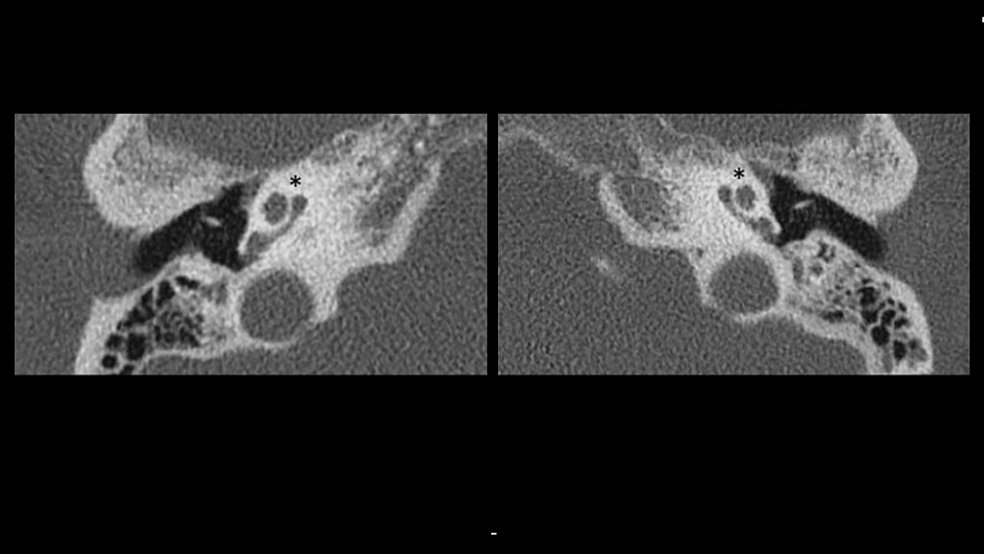
Computed tomography (CT) scan of the patient demonstrated relatively normal bony inner ear anatomy bilaterally. (*) denotes normal anatomy of the cochlea.

**Figure 3:**
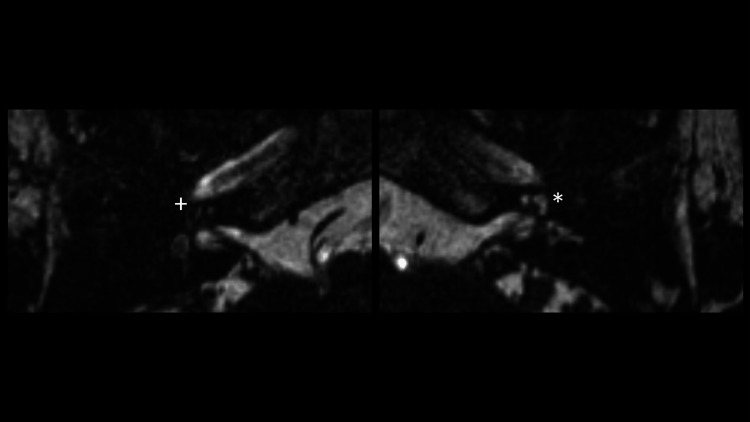
Magnetic resonance imaging (MRI) scan of the patient demonstrated a complete loss of T2 fluid signal on the right (+) and partial loss of T2 fluid signal within the basal turn of the cochlea on the left (*).

A computed tomography (CT) scan and magnetic resonance imaging (MRI) was performed (Figures 2, 3). On the right, there was evidence of complete labyrinthitis ossificans with a complete absence of a T2-weighted fluid signal within the right cochlea and labyrinth. On the left, there was a partial loss of fluid signal within the basal turn of the cochlea. Past medical history included obesity and apixaban for atrial fibrillation. 

**Figure 4:**
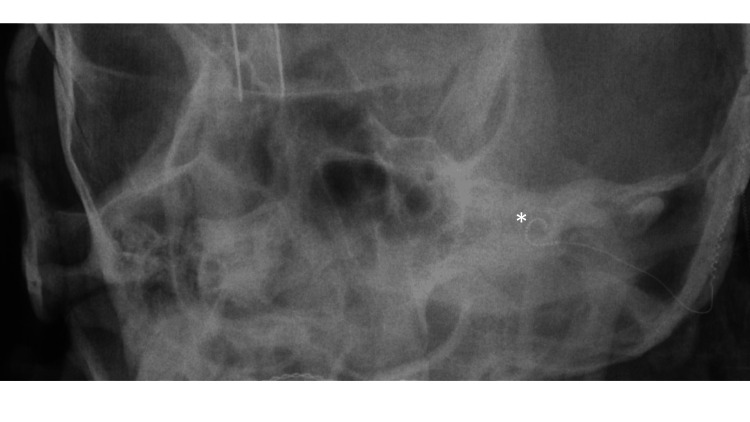
Intraoperative modified Stenver’s view X-ray demonstrating complete insertion of the cochlear implant electrode array into the cochlea (*).

The patient was counseled about the risks of surgery and the possibility of inability to achieve open set word discrimination given the degree of fibrosis and ossification of the cochlea. A canal wall down mastoidectomy with blind sac closure was performed to ensure adequate access, given the possible need for a drill-out procedure. Although the round window had been obliterated due to otosclerosis, a partial drill-out was performed. A moderate amount of fibrosis was encountered within the basal turn of the cochlea that was gently removed with a 0.4 and 0.6 mm stapes rasp. A Cochlear Nucleus CI612 cochlear implant with Contour Advance electrode was placed, and an intraoperative X-ray confirmed complete insertion (Figure 4). Post-operatively, the patient had excellent improvement in her word discrimination. One month after implantation, the patient’s consonant-nucleus-consonant (CNC) word score was 56%. Two months after implantation, her CNC score was 75%, and her Az-Bio score was 73%.

## Discussion

In this report, we present a case in which the development of meningitic labyrinthitis occurred two decades after splenectomy was performed, resulting in bilateral profound deafness. The patient’s hearing loss was treated with cochlear implantation, which was complicated by the presence of labyrinthitis ossificans. Globally, the incidence of splenectomy is 6.4-7.1 per 100,000 people per year, with trauma (25%) and hematological disorders (25%) being the most common indications [10]. Patients with absent or reduced splenic function are at risk for infection and thromboembolism. The factors responsible for an increased incidence of infection following a splenectomy include insufficient opsonizing filter function of the spleen, delayed and impaired production of immunoglobulin (Ig), and lack of splenic macrophages. Splenectomy carries a lifelong increased risk of infections, but the risk is particularly high for certain patients based on the indication for splenectomy, age at the time of splenectomy, interval since splenectomy, underlying comorbidities, immunocompromised status, and prior episode of sepsis [11].

Animal studies indicate that the sequence of events in labyrinthitis ossificans begins with inflammation that progresses to fibrosis and ossification, and further that ossification of the cochlear can continue over the years [12,13]. Histopathological analysis of temporal bone specimens has demonstrated that fibrosis can be identified as early as 1.5 weeks after meningitic labyrinthitis [14]. Labyrinthitis ossificans presents several intraoperative challenges for the CI surgeon, precluding electrode insertion via a round window or cochleostomy approach and requiring drilling of the cochlear basal turn or the use of a split electrode array [15-17]. Post-operative outcomes in CI recipients are more variable in patients with labyrinthitis ossificans. While audiologic outcomes can be adequate in the setting of cochlear fibrosis and ossification, the timing of implantation may influence postoperative outcomes [14,18,19].

## Conclusions

This patient’s history was unusual in that her splenectomy had been performed many years prior, and she was vaccinated against common encapsulated bacteria. Further prospective studies are needed to identify the causative organisms that can result in meningitic labyrinthitis despite adequate antibody titers. Prompt identification of potential neurological sequelae of septic meningitis, such as hearing loss, is critical to avoid a delay in effective surgical treatment.
